# TGF-β Concentration in Breast Milk is Associated With the Development of Eczema in Infants

**DOI:** 10.3389/fped.2018.00162

**Published:** 2018-06-01

**Authors:** Yoshinori Morita, Eduardo Campos-Alberto, Fumiya Yamaide, Taiji Nakano, Hidenori Ohnisi, Minako Kawamoto, Norio Kawamoto, Eiko Matsui, Naomi Kondo, Yoichi Kohno, Naoki Shimojo

**Affiliations:** ^1^Department of Pediatrics, Graduate School of Medicine, Chiba University, Chiba, Japan; ^2^Department of Pediatrics, Graduate School of Medicine, Gifu University, Gifu, Japan

**Keywords:** TGF-β, breast milk, infants, allergy, eczema

## Abstract

**Background:** Transforming growth factor (TGF)-β in breast milk is crucial for mucosal immune system in the neonatal period. We hypothesized that the level of exposure to TGF-β from breast milk in the first month of life is related to the development of eczema later in life. Thus, the present study investigated whether changes in TGF-β levels between colostrum and mature milk are associated with such occurrence in a birth cohort study.

**Methods:** Colostrum and 1-month breast milk samples were collected from mothers who participated in our birth cohort study. TGF-β1 and TGF-β2 levels in breast milk were measured using a commercial ELISA kit. The development of eczema in the first 6 months after birth was assessed based on parent's response to a questionnaire. Levels of TGF-β1 and TGF-β2 were compared in breast milk from mothers of infants with and without eczema.

**Results:** In children with eczema, TGF-β1 levels were higher in colostrum, but lower in 1-month milk. A lower TGF-β1 ratio (1-month milk/colostrum) was related to the development of eczema during the first 6 months of life. There was no difference in TGF-β2 ratio (1-month milk/colostrum) between eczema group and control group.

**Conclusions:** Concentration of TGF-β1 but not TGF-β2 in breast milk during the first month after birth may be associated with eczema later in life. Factors that increase TGF-β1 levels in breast milk may play a role in preventing allergic disease.

## Introduction

Breast milk contains a wide variety of immune mediators, such as secretory immunoglobulin A, soluble CD14, and transforming growth factor (TGF)-β (1). These mediators are crucial for mucosal immune system in the neonatal period.

TGF-β may play a role in preventing allergic diseases. In animal models, the oral administration of TGF-β1 maintained the biological activity in the intestinal mucosa and enhanced the induction of oral tolerance (2). It has also been reported to inhibit inflammation in the intestinal epithelium and systemic production of interleukin-6 and interferon-γ, reducing the incidence of necrotizing enterocolitis (NEC) (3). In contrast, developmental defects rather than immunological dysregulation were observed in TGF-β2- and TGF-β3-deficient mice (4, 5).

Several studies have been conducted to address whether the levels of TGF-β in breast milk are related to the development of allergic diseases. Although a review article reported TGF-β in breast milk is an effective cytokine in preventing allergic disease (6), the results of several other studies were controversial (7–9). Hence, we hypothesized that the amount of TGF-β received in the first month of life is important in the prevention of allergy, and in the present study we investigated whether changes in the TGF-β cytokine level in breast milk from colostrum to mature milk were related to the future development of eczema in infants.

## Materials and methods

### Study design

This study is part of a birth cohort study of 500 newborn infants, which was conducted in Kawatetsu-Chiba Hospital from January 2007 to May 2008. The study design has been described in detail elsewhere (10). Briefly, all participants received a questionnaire when their infants were born. Data on parental allergic disease and various exposures were obtained. The parents also answered questionnaires on symptoms related to eczema in their infant at 1, 4, and 6 months of age. Eczema was defined as itchy skin rashes lasting >2 months in 6-month-old children.

Infants who presented with eczema at 6 months were included in the eczema group. As for the control group, we randomly selected infants who did not have eczema at 6 months. Both groups were matched in terms of feeding methods and history of atopic dermatitis or other allergic diseases in the mother (asthma, allergic rhinitis, food allergy, and pollen hypersensitivity). Levels of TGF-β1 and TGF-β2 in breast milk were measured in both groups.

The study protocol was approved by the Ethics Committee of Chiba University Graduate School of Medicine (No. 570). All parents provided written informed consent.

### Breast milk collection and processing of breast milk

Colostrum samples were collected within 5 days post-partum and mature milk samples were collected 1 month after delivery. Each sample was frozen within 12 h of collection and kept at −80°C. After thawing, the samples were centrifuged at 10,000 g for 10 min, the cellular debris and fat layer were discarded, and the clear middle layer was used for analyses.

### Measurements of TGF-β1 and TGF-β2 in breast milk

TGF-β1 and TGF-β2 levels in breast milk were measured with the Quantikine Human TGF-β1 and TGF-β2 Immunoassays (R&D Systems Inc, MN, USA). Activation of TGF-β in breast milk was performed using 1N HCl and neutralized with 1N NaOH. The sensitivity threshold for both the TGF-β1 and TGF-β2 assays were 31.25 pg/mL. TGF-β1 levels below 31.25 pg/mL were also measured since we could confirm linearity with sequential dilution down to 1.95 pg/mL. We therefore defined 3.9 pg/mL as the detection limit of the TGF-β1 assay in this study. Since the clear middle layer was diluted 40 times in the neutralization step for TGF-β1 analysis, 156 pg/mL was defined as the detection limit. Undetectable levels of TGF-β1 were set as half the value of the minimum detectable level at 78 pg/mL. The clear middle layer was diluted 7.8 times in the neutralization step for TGF-β2 analysis, 243.75 pg/mL was defined as the detection limit. Undetectable levels of TGF-β2 were set as half the value of the minimum detectable level at 121.9 pg/mL.

### Statistical analysis

Differences in the characteristics of the participants were assessed by using chi-squared test or Fisher's exact test and expressed as p values. Mann-Whitney *U*-test was used to compare TGF-β levels in both colostrum and mature milk and TGF-β ratio (1-month milk/colostrum) between eczema and control group. Changes in TGF-β levels from colostrum to mature milk was assessed with the Wilcoxon signed rank test. Adjusted logistic regression analyses were conducted to evaluate the association between eczema and TGF-β ratio. The TGF-β ratio was categorized into tertile as low, medium, or high levels.

For all analyses, *p* < 0.05 was considered statistically significant. Statistical analysis was performed with SPSS software ver. 11.0 (SPSS INC., Chicago, IL, USA).

## Results

### Study subjects

Fifty-one infants developed eczema at 6 months of age. Because of the lack of mother's milk, 8 infants with eczema were excluded from the study, leaving 43 subjects for final analysis. As controls, 53 children without eczema were randomly selected. Table 1 shows the characteristics of the participants. The only difference between the two groups was gender proportion, with a higher number of girls in the control group.

**Table 1:** Characteristic of the participants.

	**Eczema group (*n* = 43)**	**Control group (*n* = 53)**	***p*-value**
Sex (boy: girl)	30:13	21:32	< 0.05
Father allergy	26	40	0.12
Mother allergy	28	38	0.49
Mother atopic dermatitis	11	12	0.74
Mother smoking	2	5	0.37
Pet	11	16	0.62
Feeding methods			
Mixed	22	30	0.59
Breast feeding			
0–1 months	6	3	0.15
1–5 months	4	4	0.52
≥ 6 months	11	16	0.62

### TGF-β levels in colostrum and infant eczema

In colostrum, TGF-β1 was undetectable in 29 samples of the control group, but only in 6 samples from the eczema group. TGF-β2 was detectable in all 96 samples. Undetectable TGF-β1 level in colostrum was set at 78 pg/mL.

**Figure 1:**
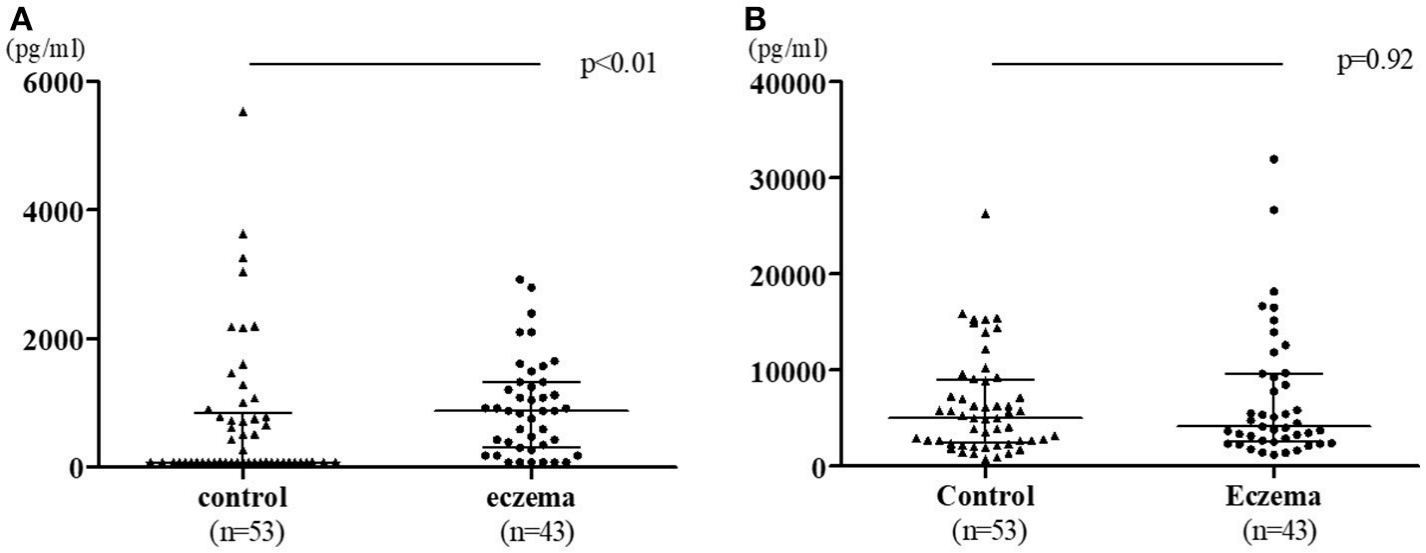
TGF-β1 **(A)** and TGF-β2 **(B)** levels in colostrum in eczema group (43 subjects) vs. control group (53 subjects). Data is shown with medians and interquartile ranges.

Figure 1 shows the comparison of TGF-β1 and TGF-β2 levels in colostrum in both groups. TGF-β1 levels were significantly higher in the eczema group with a median 878.8 pg/mL (range 78–2,921 pg/mL) than that of control group (median: 78 pg/ml, range 78–5,538 pg/ml, *p* < 0.01, Figure 1A), while the levels of TGF-β2 was not significantly different between eczema group and control group (median: 4,129 pg/mL, range 1,175–31,900 pg/mL; and median: 4,977 pg/mL, range 721.2–26,260 pg/mL, respectively, *p* = 0.92, Figure 1B).

### TGF-β levels in 1-month milk and infant eczema

In 1-month milk, TGF-β1 were undetectable in 19 samples from the control group and 24 in the eczema group. TGF-β2 were undetectable only in 4 samples of the eczema group. Undetectable levels of either TGF-β1 in 1-month milk was also set at 78 pg/mL. Undetectable levels of TGF-β2 in 1-month milk was also set at 121.9 pg/mL.

**Figure 2:**
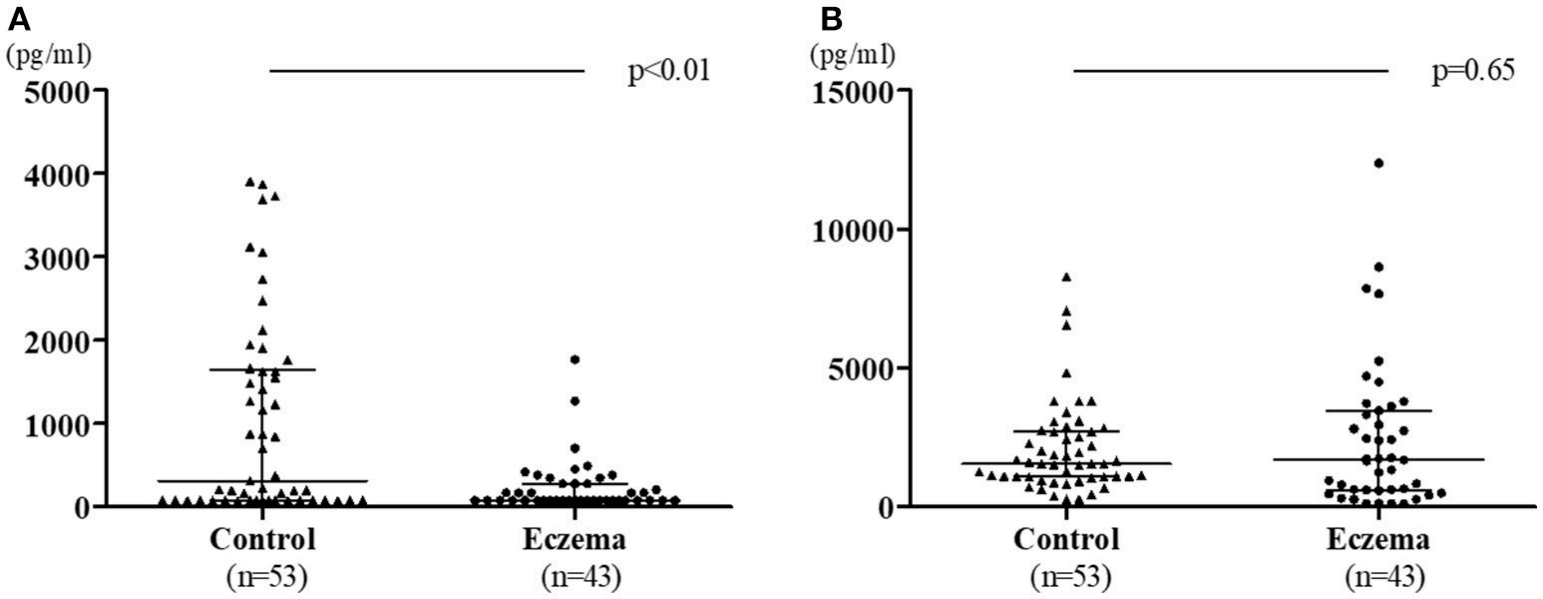
TGF-β1 levels **(A)** and TGF-β2 levels **(B)** in 1-month milk in eczema group (43 subjects) and control group (53 subjects). Data is shown with medians and interquartile ranges.

Figure 2 shows the comparison of TGF-β1 and TGF-β2 levels in 1-month milk in both groups. In contrast to colostrum, TGF-β1 levels in the eczema group was lower than those in the control group (median: 78 pg/mL, range 78–1,768 pg/mL; and median: 311.2 pg/mL, range 78–3,903 pg/mL, respectively, *p* < 0.01, Figure 2A), while there was no significant difference in the TGF-β2 levels between eczema group and control group (median:1,692 pg/mL, range 121.9–12,370 pg/mL; and median 1,550 pg/mL, range 255.2–8,273 pg/mL, respectively, *p* = 0.65, Figure 2B).

### TGF-β levels ratio and eczema in infants

**Figure 3:**
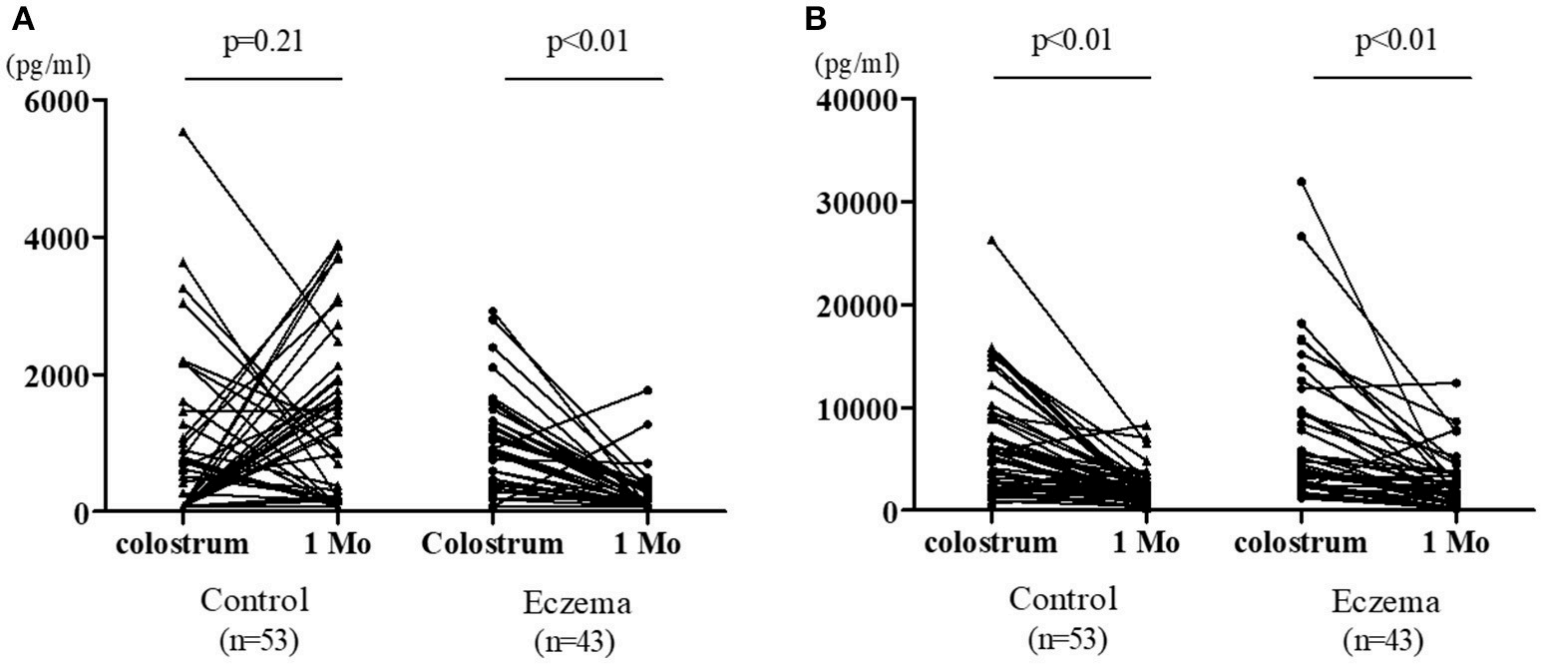
Individual values of TGF-β1 **(A)** and TGF-β2 **(B)** in colostrum and 1-month milk in eczema group (43 subjects) and control group (53 subjects).

Figure 3 shows the change in TGF-β levels in colostrum vs. 1-month milk in both groups. TGF-β1 concentration did not decrease in the control group (*p* = 0.21); however, there was a significant reduction in the eczema group (*p* < 0.01, Figure 3A). TGF-β2 concentration significantly decreased in both groups (*p* < 0.01, Figure 3B).

**Figure 4:**
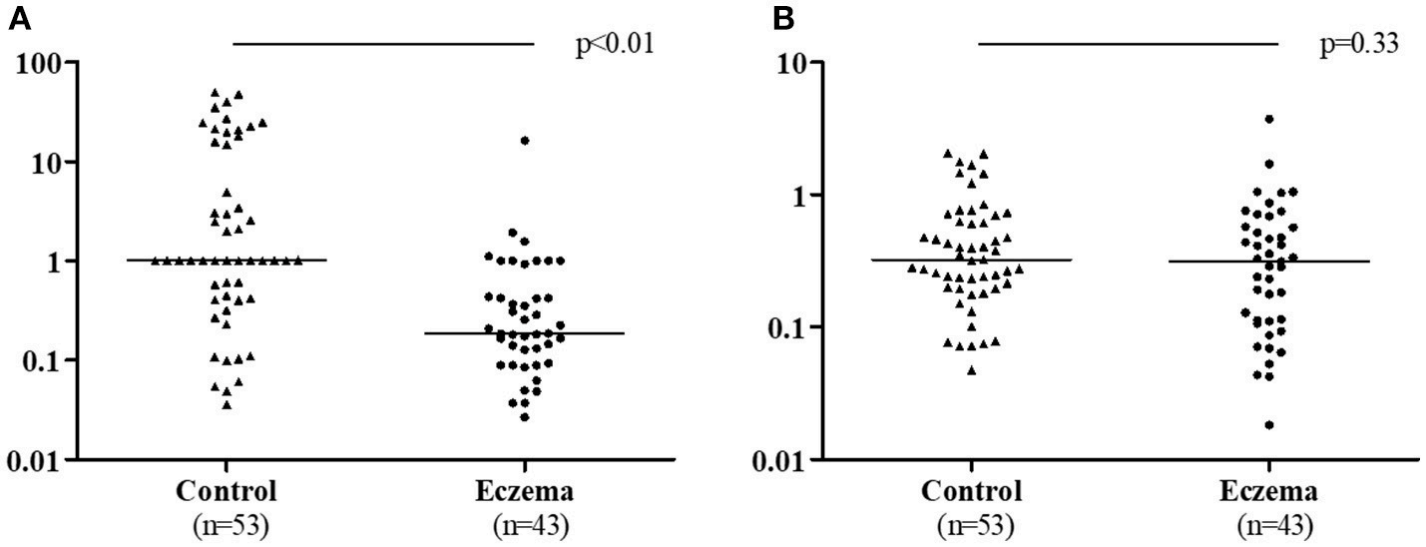
TGF-β1 ratio (1-month milk/colostrum) **(A)** and TGF-β2 ratio (1-month milk/colostrum) **(B)** in eczema group (43 subjects) and control group (53 subjects).

TGF-β1 ratio (1-month milk/colostrum) was lower in the eczema group than in the control group (*p* < 0.01, Figure 4A). The TGF-β2 ratio was not statistically different between the two groups (*p* = 0.33, Figure 4B).

### Multivariable analysis

Table 2 describes the results of multivariate analysis with logistic regression. The risk of eczema was inversely related to TGF-β1 ratio.

**Table 2:** Multiple regression analysis for the effect of TGF-β ratio on eczema.

**Factor**		**Adjusted odds ratio**	**95% CI**			***p*-value**
Sex (Male)		2.084	0.774	~	5.610	0.146
Mother allergy		0.560	0.185	~	1.699	0.306
Breast feeding by 1 month		1.203	0.444	~	3.288	0.711
**TGF-**β**1 RATIO (1-MONTH/COLOSTRUM)**						
Low	0.0267–0.224	1				
Middle	0.229–1	0.161	0.049	~	0.534	< 0.01
High	1.013–49.585	0.035	0.006	~	0.188	< 0.01
**TGF-**β**2 RATIO (1-MONTH/COLOSTRUM)**						
Low	0.0182–0.214	1				
Middle	0.231–0.464	0.848	0.250	~	2.878	0.792
High	0.473–3.731	2.959	0.702	~	12.467	0.139

*Logistic regression models adjusted for sex, maternal history of allergies, breast feeding by 1 month and TGF-β ratio*.

### Effect of maternal allergic history on TGF-β levels in breast milk

We also compared TGF-β1 and TGF-β2 levels in both colostrum and 1-month milk in allergic mothers. The levels of TGF-β1 and TGF-β2 in colostrum were not significantly different between allergic mothers (median: 511.1 pg/mL, range 78–5,538 pg/mL; and median: 4,826 pg/mL, range 721.2–31,900 pg/mL, respectively, *p* = 0.56), and in non-allergic mothers (median: 511.1 pg/mL, range 78–2,799 pg/mL; and median: 4,553 pg/mL, range 1,402–16,650 pg/mL, respectively, *p* = 0.55), neither were their concentrations in 1-month milk in allergic (median: 169.1 pg/mL, range 78–3,903 pg/mL; and median: 1,678 pg/mL, range 121.9–12,370 pg/mL, respectively, *p* = 0.4) and non-allergic mothers (median: 183.4 pg/mL, range 78–3,119 pg/mL; and median: 1,129 pg/mL, range 121.9–7,852, respectively, *p* = 0.28).

## Discussion

We demonstrated that principally a lower TGF-β1 ratio (1-month milk/colostrum) was related to later eczema in infants.

Reports on the relationship between TGF-β in breast milk and onset of allergic diseases are controversial. Some studies have reported that neither TGF-β1 nor TGF-β2 was associated with the onset of allergic disease (7–9, 11), while others have demonstrated such an association (12–14). In the present study, we found a significant relationship between TGF-β1 levels in breast milk and the development of eczema. In the eczema group, the TGF-β1 concentration was higher in colostrum, but lower in 1-month milk. In contrast with previous reports, we measured TGF-β at multiple time points and assessed the TGF-β ratio.

The higher dose of TGF-β1 (long breast feeding and medium-high TGF-β1) in breast milk were shown to have a protective effect against wheezing (12); although the outcome was different, we saw a similar pattern with eczema in our study. Riggoti et al. reported that TGF-β1 was significantly higher in the mature milk of non-allergic mothers, and infants fed with this TGF-β1-rich breast milk had less risk of developing atopic diseases (15), further emphasizing the protective effects of this cytokine against eczema. Their results might be due to genetic causes as they had grouped their cohort based on the mother's allergic background. Levels of TGF-β1 reportedly decreases from colostrum to mature milk in urban mothers but not in rural mothers (16). These findings and our results suggest that a significant decrease in TGF-β1 in colostrum to mature milk is associated with later allergic disease.

Lower levels of TGF-β2 in colostrum were observed with *Lactobacillus reuteri* supplementation during pregnancy, leading to less sensitization in children of those mothers during the first 2 years of life (13). Nonetheless, higher levels of TGF-β2 in breast milk were reportedly related to higher risk of eczema (14). In our study, both TGF-β2 levels and 1-month/colostrum ratio for TGF-β2 were not different between eczema group and control group.

The factors influencing the levels of TGF-β in breast milk are not well known. TGF-β levels in breast milk differed between countries (17, 18), and even races in the same country (19, 20). Similar to our results, no difference was observed in the allergic history of mothers (21). There have been conflicting results between the supplementation of probiotics during pregnancy and TGF-β1 levels, with a previous study reporting a significant increase in TGF-β1 levels (22); although, other investigations did not support such a result (23–27). Further studies are needed to clarify the factors influencing the levels of TGF-β in breast milk.

As for the limitations of this study; first, in our recruitment, we had more boys in the eczema group. And second, we could not evaluate long-term outcomes. Nonetheless, the present study suggests that TGF-β1 levels in breast milk are related to the occurrence of eczema later in life.

In conclusion, the present investigation suggested that a higher 1-month/colostrum ratio for TGF-β1 is associated with a more consistent concentration or an increase of TGF-β1 across the first month, which has a protective effect in reducing the risk of eczema. Additional studies are needed to evaluate the relationship between TGF-β levels in breast milk and the development of allergic disease. Identifying factors that increase TGF-β1 levels in breast milk may contribute to preventing allergic diseases.

## Author contributions

YM wrote this manuscript and did statistical analysis. EC-A wrote this manuscript. FY, TN and NoK provided critical advice. YK and NaK conducted study. HO, MK and EM measured TGF-β. NS conducted study and wrote this manuscript.

### Conflict of interest statement

The authors declare that the research was conducted in the absence of any commercial or financial relationships that could be construed as a potential conflict of interest.
